# Mesenchymal stromal cell-derived extracellular vesicles: novel approach in hematopoietic stem cell transplantation

**DOI:** 10.1186/s13287-022-02875-3

**Published:** 2022-05-16

**Authors:** Davod Pashoutan Sarvar, Hosein Effatpanah, Parvin Akbarzadehlaleh, Karim Shamsasenjan

**Affiliations:** 1 Asadabad School of Medical Sciences, Asadabad, Iran; 2 Department of Pharmaceutical Biotechnology, Tabriz University of Medical Science, Tabriz, Iran; 3 Hematology and Oncology Research Center, Tabriz University of Medical Sciences, Tabriz, Iran

**Keywords:** Extracellular vesicles, Hematopoietic stem cells, Hematopoietic stem cell transplantation, Mesenchymal stem cells

## Abstract

Bone marrow mesenchymal stromal cells (MSCs) play a crucial role in the regulation of hematopoiesis. These cells affect the process through direct cell–cell contact, as well as releasing various trophic factors and extracellular vehicles (EVs) into the bone marrow microenvironment. MSC-derived EVs (MSC-EVs) are prominent intercellular communication tolls enriched with broad-spectrum bioactive factors such as proteins, cytokines, lipids, miRNAs, and siRNAs. They mimic some effects of MSCs by direct fusion with hematopoietic stem cells (HSC) membranes in the bone marrow (BM), thereby affecting HSC fate. MSC-EVs are attractive scope in cell-free therapy because of their unique capacity to repair BM tissue and regulate proliferation and differentiation of HSCs. These vesicles modulate the immune system responses and inhibit graft-versus-host disease following hematopoietic stem cell transplantation (HSCT). Recent studies have demonstrated that MSC-EVs play an influential role in the BM niches because of their unprecedented capacity to regulate HSC fate. Therefore, the existing paper intends to speculate upon the preconditioned MSC-EVs as a novel approach in HSCT.

## Introduction

Mesenchymal stromal cells (MSCs) as multipotent stem cells are fibroblast-like cells that could be isolated from various tissues and body fluids. Although MSCs derived from several tissues have no identical antigens but have different duties. MSCs are multipotent stem cells that can specialize into chondrogenic, adipogenic, and osteogenic lineage cells. They are one of the most vital components of the bone marrow microenvironment, which plays a significant role in homing, proliferation, and differentiation of hematopoietic stem cells (HSCs) [1–3]. MSCs perform their functions by cell-to-cell communication similarly direct cell contact and releasing of soluble growth factors [4]. MSCs with the potential to differentiate into damaged cells have an irreplaceable role in tissue repairing and find a special place in regenerative medicine [5, 6]. As well as secretion of cytokines leads to immunosuppression, thus playing a role in the suppression of graft versus host disease (GVHD) [7, 8].

Nowadays, co-transplantation of MSCs with HSCs finds significance in the clinical decision for treating patients with hematological disorders because they can support the HSC niche in the bone marrow (BM) [9]. Several studies have recently proved the supportive role of MSCs in hematopoiesis through direct cell-to-cell contact, cytokines secretion, and soluble growth factors [10–12]. In addition, MSCs have supportive effects on the autologous engraftment of HSCs in animal models [13]. But due to some complications, infusion of MSCs in hematopoietic stem cell transplantation (HSCT) is limited. More recently, several studies have revealed that MSC-derived extracellular vesicles (MSC-EVs) alter the function of neighboring or distant cells because they carry multiple bioactive substances such as lipids, proteins, miRNAs, piRNAs, and siRNAs [14–17]. MSC-EVs have supportive roles in repairing tissue damages (e.g., BM, heart, lung, brain, and liver) [18] and suppressing inflammatory responses [19]. Also, the immunosuppression capacity of MSC-EVs has been reported [20, 21].

The investigation of MSC-EV applications in HSCT is an attractive field of regenerative medicine because these vesicles mimic the corresponding effects of MSCs by fusion with the HSC membrane in the BM. In this review, we discuss what is comprehended about the interaction effects between extracellular vesicles (EVs) secreted by MSCs and HSCs, also its applications as novel frontiers in HSCT.

### The characteristics of MSCs

MSCs mainly present in the bone marrow and contain 0.001–0.01% of the total nucleated cells. In addition, MSCs were isolated from umbilical cord blood (UCB), placenta, Wharton’s jelly, brain, liver, adipose tissue, spleen, thymus, lung, dental pulp, palatine tonsils, and peripheral blood [22]. International Society for Cellular Therapy (ISCT) enacts the minimum criteria to define MSC: (1) Plastic‐adherence potency in standard culture conditions, (2) Presence of CD105 (SH2), CD73 (SH3/4), CD90 markers and lack expression of CD45, CD34, CD14, and HLA‐DR markers and (3) Potency of multi-lineage differentiation into adipocytes, osteoblasts, and chondroblasts in vitro [3, 23]. The latter property has made MSCs an initial choice for tissue regeneration in the treatment of many diseases [24–26]. Apart from direct cell-to-cell contact, MSCs with secretion of various soluble mediators and cytokines play an essential role in proliferation and differentiation of HSCs in the bone marrow [27], as well as MSCs produce stromal cell-derived factor 1 (SDF1), also known as CXCL12 (C-X-C Motif Chemokine Ligand 12), which has a significant role in homing, quiescence, and repopulating function of HSCs [27, 28]. Hence, controlling of balance between HSC quiescence, self-renewal, and differentiation into mature cells depend on signals received by their local microenvironment.

### The characteristics of MSC-EVs

Microvesicles (MVs) and exosomes (EXs) are MSCs-derived EVs. These particles are different from each other based on origin and size. EXs have 30–200 nm in diameter and originate from the membrane of late endosomes, while MVs are in the size range of 200–1000 nm in diameter that derives from the cell membrane [29, 30].

Multiple cell types secrete EVs such as stem cells, dendritic cells, lymphocytes, mast cells, epithelial cells, and tumor cells [31, 32]. These vesicles are found in body fluids such as blood, urine, breast milk, saliva, bronchoalveolar lavage (BAL) fluid, amniotic fluids, cerebrospinal fluid (CSF), and seminal fluid [33, 34].

Cell-derived EVs contain membrane-associated proteins such as tetraspanins (e.g., CD9, CD63, CD81, and CD82), cytoskeletal proteins (e.g., actin, syntenin, and moesin), heat-shock proteins (e.g., Hsp70, Hsp90, Hspa8, and Hsp60), and proteins implicated in multivesicular body synthesis (Alix and TSG101). In addition, Mesenchymal stem cell-derived extracellular vesicles (MSC-EVs) express MSC markers such as CD105, CD90, CD29, CD73, CD44, and KIT (CD117) but do not show hematopoietic antigens (e.g., CD11b or CD34, CD45, CD79 or CD19, and HLA-DR) [35, 36]. EVs include trophic factors, cytokines, and small RNAs (microRNA, piRNA, and siRNA) [14, 37]. Recently, 10,520 miRNA entries were submitted from researches based on Vesiclepedia database (http://microvesicles.org). Production and release of these vesicles are affected by various chemical, environmental and mechanical stimulants, including gamma-irradiation, statins, heparanase, calcium ionophores, hypoxia (low O2), and acidosis conditions [3]. Granulocyte colony-stimulating factor (G-CSF) stimulates the release of EXs from hematopoietic progenitor cells (HPCs) [38].

The content of these vesicles relied heavily on the cell life-span, oxidative stress, and environmental signals [38, 39]. As well, there are reports that EXs from aged cells are rich in miR-183-5p. These EXs cause a reduction of proliferation and differentiation of young BM stromal cells [40].

There are many methods for the isolation of EVs. Differential ultracentrifugation combined with sucrose density gradients is the most frequently used method for the MSC-EVs isolation from cell culture supernatants and body fluids. Additional isolation methods are ultrafiltration, high-performance liquid chromatography (HPLC), size-exclusion chromatography (SEC), precipitation using volume excluding polymers (e.g., polyethylene glycols), affinity purification using specific antibodies against CD9, CD63, CD81, and CD82, tangential flow filtration (TFF), magnetic bead isolation and fluorescence-activated cell sorting (FACS) [41, 42].

Next to isolation, these vesicles must be identified and stored in optimal conditions until therapeutic applications. EVs are identified by two or three of the following methods. These methods are transmission electron microscopy (TEM), scanning electron microscopy (SEM), cryo-electron microscopy (Cryo-EM), dynamic light scattering (DLS), nanoparticle tracking analysis (NTA), atomic force microscopy (AFM), enzyme-linked immunosorbent assay (ELISA), western blotting or flow cytometric examination [3, 10].

MSC-EVs affect the various type of recipient cell types, especially HSCs in the BM stroma. Some researchers reported the regenerative role of these vesicles in damaged tissues [43–45]. These vesicles play a crucial role in cellular functions such as tissue hemostasis, cell cycle regulation, cell migration, and hematopoiesis which are mainly mediated by miRNAs [46]. MSC-EVs suppress inflammatory reactions and modulate immune system responses. MSC-EVs suppress inflammatory reactions and modulate immune system responses. MSC-EVs play a critical role in inhibiting tumor cell progression, metastasis, and angiogenesis [47]. It has been proven direct effectors such as WNT, β-catenin, and Hedgehog in MSC-EVs play crucial roles in stem cell biology [48].

### The potential role of MSCs in hematopoietic system

MSCs incorporate in tissue regeneration with differentiation potency to stroma cells. MSCs have been shown to support the expansion and proliferation of HSCs and their progenitors. Additionally, these cells inhibit HSCs apoptosis [1, 3]. Inhibition of TGF-signaling pathway with SiRNA targeting TGF-RII in CD34+ cells and their co-culture with MSCs increase HSC expansion [49].

In co-culture conditions, MSCs in combination with low oxygen pressure (5% O2) improve the expansion and homing capacities of HSCs [50]. In addition, MSCs play a positive role in the differentiation of HSCs in vivo and in vitro. MSCs have suppressor effects on the erythroid differentiation in the K562 cell lines [51]. Perucca et al. demonstrated that MSCs have an essential function in regulating proliferation and erythroid differentiation of CD34+ stem cells [52].

In several studies, the supportive effects of MSCs on myeloid differentiation of HSCs have been proven [53, 54]. Molaeipour et al. demonstrated that MSCs have a collateral role in the monocytic differentiation of U937 cell lines [55]. In another study, it has been shown that BM-MSCs promote the granulocytic differentiation of HL-60 cell lines [56]. Another research showed that co-infusion of MSCs enhances myeloid and megakaryocytic differentiation of HSCs [57]. Generally, MSCs have a significant role in the coordination of normal hematopoiesis and the ratio of myeloid to erythroid precursors (M/E) in the bone marrow. These results, therefore, provide MSCs as an effective adjuvant for HSCT.

In recent years, various researches revel that co-transplantation of HSC-MSC increases the success rate of HSCs engraftment [58, 59]. In general, the engraftment of HSCs relied on HSC homing increment and suppression of GVHD. From the point of paracrine effects, MSCs secrete stem cell factor (SCF), SDF-1, and FMS-like tyrosine kinase 3 (Flt-3) ligand, which enhances HSC homing to BM. In addition, MSCs modulate innate and adaptive immune responses via the production of soluble factors such as indoleamine 2,3-dioxygenase (IDO) and PGE2, and polarization of T cells to Treg cells (CD25+ FoxP3+) [60, 61]. Furthermore, MSCs facilitate the differentiation of monocytes into M2 macrophages that produce immunosuppressive cytokines such as IL-10, resulting, these cells playing a critically important role in preventing GVHD development [61].

### The potential role of MSC-EVs in hematopoietic system

In addition to MSCs, MSC-EVs have a crucial role in determining HSC fate. Limited studies have been performed to clarify MSC-EVs and HSC interactions. One study has demonstrated that MSC-MVs support the proliferation of primary CD34+ cells in vitro [62]. Two studies showed that vesicles derived from MSCs prevent HSCs apoptosis and induce engraftment of them by increasing Cysteine-X-cysteine (CXC) motif chemokine receptor type 4 (CXCR4) and chemokine expressions [14, 63]. Another study revealed that bioactive molecules in MSC-EVs modulate gene expression of HSCs to enhance HSCs homing in the BM niche [64]. MSC-MVs enhance the proliferation of the umbilical cord blood-derive HSCs in vitro. In addition, adding MSC-MVs into the MSCs and HSCs co-culture system enhanced HSC proliferation [62, 65]. Morhayim et al. have found that EVs derived from osteoblasts increase the proliferation of UCB-derived CD34 + cells in vitro and *Vivo* [66]. Furthermore, Preciado et al. showed that EVs derived from MSCs increase the clonogenic capacity of CD34^+^ cells via increasing *BIRC2*, *BIRC3*, and *NF-κB* expression. While proapoptotic genes such as* CASP3* and* CASP6* were downregulated. In addition, CD44, a significant molecule in homing and engraftment of HSCs, upregulation was reported [67].

It has been revealed that BM-MSC-derived vesicles restore radiation-induced bone marrow impairment by augmentation in HSC proliferation and inhibition of DNA damages [68].

It has been reported that infusion of MSC-EVs alone can recover hematopoiesis in irradiated mice without hematopoietic engraftment [69]. Also, another study has shown that human induced pluripotent stem cells-derived EVs (hiPSC-EVs) increase the reconstitution capacity of HSCs [70]. Treatment of HSCs/HPCs with G-CSF increases the levels of miR-126 inside the EXs. miR-126 has a role in the detachment and mobilization of HSCs/HPCs into the peripheral blood via inhibition of expression of vascular cell adhesion molecule-1 (VCAM-1) [38]. G-CSF is considered as a helpful adjunct to the recovery of hematopoiesis following radiotherapy in HSCT. Since the G-CSF treatments are costly and complicated to produce, therefore, MSC-EVs can be a benevolent replacement for G-CSF in HSCT [71].

MicroRNAs, important compositions of EVs, have a critical effect on the gene expression profile of HSCs. The majority of miRNAs play a crucial role in the proliferation and differentiation of HSCs via regulation of the Wnt/β-catenin signaling pathway [62]. The miRNA-125 family members, including miR-125a, miR-125b1, and miR-125b2, are essential for the self-renewal and differentiation of HSCs [72]. Also, previous studies showed that miRNA-125a strongly increases the proliferation of HSCs and progenitors but reduces their apoptosis [73, 74]. In this regard, a recent study showed that miR-125a enriched in the EVs derived from adult BM-MSCs has a principal role in ex vivo proliferation of HSCs/HPCs by regulation of apoptosis [75]. In addition to miR-125a, miR-21 is involved in hematopoiesis [62].

**Fig. 1:**
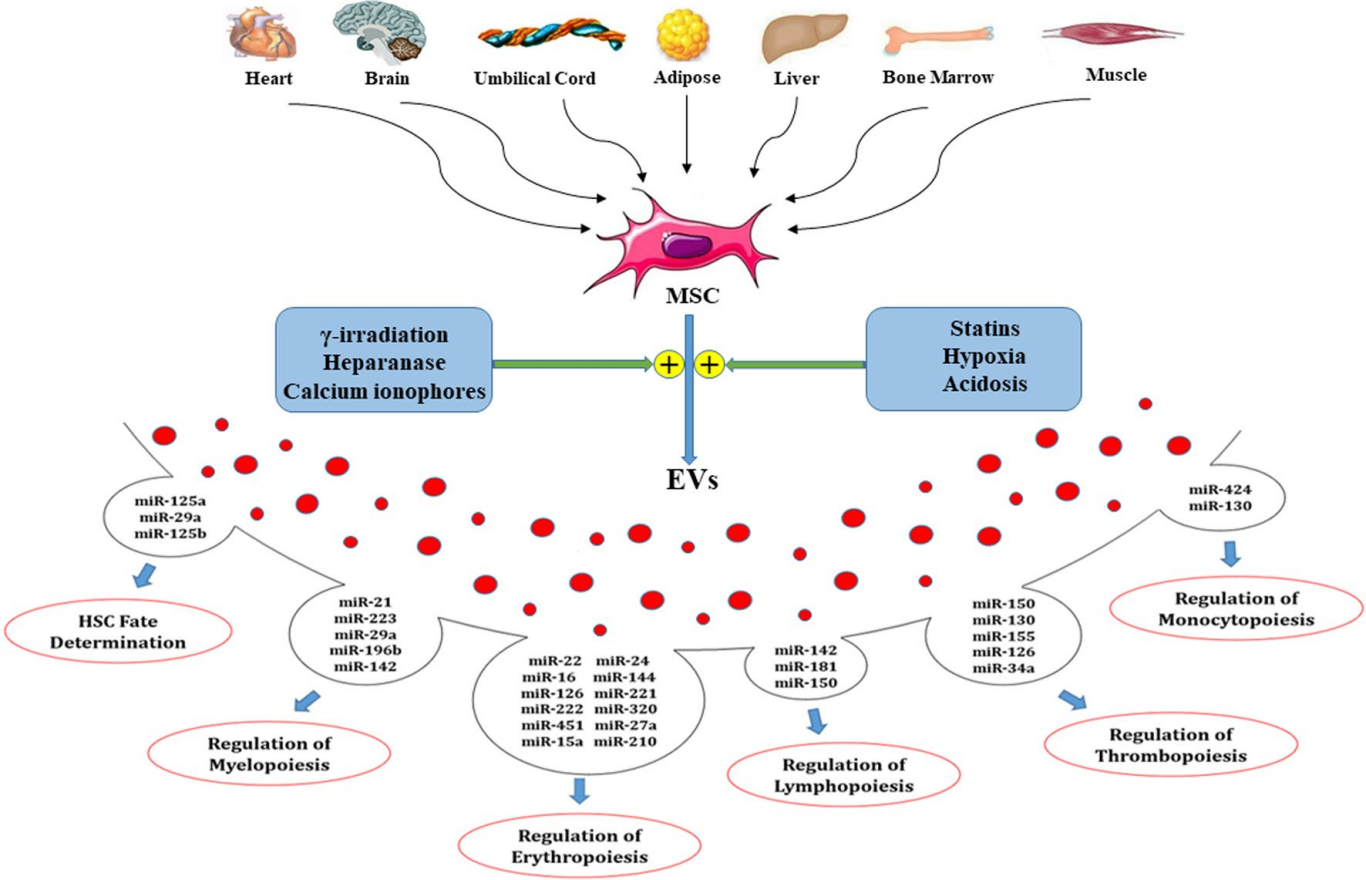
Mesenchymal stem cells (MSCs) derived from different sources under various chemical, environmental and mechanical stimulants, including gamma-irradiation, statins, heparanase, calcium ionophores, hypoxia and acidosis conditions are able to release extracellular vesicles (EVs) that are enriched with trophic substances. These cargos are transferred to recipient cells and affect the functions of them. MicroRNAs as important compositions of EVs have an important role in proliferation and differentiation of HSCs

miRNA-223 acts as a positive regulator of granulopoiesis by increasing the *Mef2c* gene expression but repressing the erythroid differentiation transcription factor *NFI-A*. In erythropoiesis, *GATA-1* and *GATA-2*, two essential lineage transcription factors, are regulated by miRNA-144 and miRNA-451 [72]. Another miRNA that enriched MSC-EVs is miRNA-21. miRNA-21 preserves HSCs from irradiation-induced damage via activating the *NF-κB* pathway and regulation of HSC hemostasis [76]. In addition, miRNA-21 plays a suppression role in myelopoiesis by targeting the *Smad* pathway [77]. MiRNA-196b are released by MSC-EVs directly targets *HOXA9*/*MEIS, therefore, play a positive role in myelopoiesis* [78, 79]. It has been indicated that growth factor independent-1 (*Gfi-1*) as granulocytic differentiation transcription factor regulates the expression of miR-21 and miR-196b [80]. Goloviznina et al. in 2016 showed that MSC-EVs induce differentiation of HSC progenitors via MyD88-dependent TLR4 signaling. Goloviznina et al. 2016 showed that MSC-EVs induce differentiation of HSC progenitors via MyD88-dependent TLR4 signaling. MSC-EVs by increasing the number of myeloid progenitors have a supportive role in the myeloid differentiation of HSCs [81]. MSC-derived microvesicles that enriched with miR-424, miR-150, and miR-181 regulate differentiation of monocyte, B, and T lymphocyte lineages, respectively [62]. Therefore, the balance between the suppressor and inducer miRNAs has a quintessential role in determining HSC fate (Fig. 1).

Exosomal miRNA-486 derived from the supernatants of TF-1 cell culture media increases hypoxia-induced erythroid differentiation of TF-1 and CD34+ cells by inhibiting *Sirt1* gene expression [82]. Additionally, another study showed that miRNA-486 expression increased during erythroid differentiation of both chronic myeloid leukemia (CML) progenitor and normal CD34+ cells [83]. In contrast to these studies, we have previously revealed that MSC-EVs have inhibitory effects on erythroid differentiation of umbilical cord blood-derived CD34+ cells [84]. Hence, MSC-EVs play a significant role in normal hematopoiesis and M/E ratio regulation in the bone marrow.

### MSCs versus MSC-EVs in HSCT

Nowadays, HSCs are co-transplanted with MSCs in the treatment of hematological disorders. It mostly takes root in the supportive role of MSCs in hematopoiesis. Although MSCs have been shown to reduce the risk of acute GVHD (aGVHD) in coadministration with HSCs during bone marrow transplantation (BMT), despite these benefits, several studies reported MSC disadvantages for cell therapy. These cells severely suppress the immune system, which increases the risk of infections, especially in children. MSCs could be carriers for several microorganisms such as* Parvovirus B19* (B19),* Cytomegalovirus* (CMV),* Herpes Simplex-1* (HSV-1), and* Mycoplasma hyorhinis* that has an anti-proliferative effect on MSCs [85, 86].

Could be safe and non-infectious however may lead to suppression of the immune system of an infected recipient [86, 87].

MSCs harboring* Mycoplasma hyorhinis* have an inhibitory effect on the proliferation of lymphocytes, and its transplantation could lead to induction of infection risk [87]. Therefore, this can be a barrier to the suppression of GVHD after HSCT.

MSCs are involved in tissue regeneration, especially bone marrow that can be utilized in aplastic anemia (AA) and bone marrow failure syndrome (BMFS) [88]. MSC-EVs have miR-335 that promote bone regeneration through VapB and the Wnt/β-catenin pathway [89]. Gholampour et.al report manifested that MSC‐EVs improve BM deficiency and attenuate AA development by modifying immune responses in a mouse model of AA. They revealed that miR‐126a, miR‐146a, miR199a, and miR‐223 in MSC‐EVs have an inhibitory activity on the proliferation of T cells, as well as the IFN‐γ and TNF‐α cytokine expressions [90].

But ectopic differentiation and malignant transformation of MSCs seem to make this trend a problem. Some studies have demonstrated a malignant growth of tumor cells induced by MSC infusion [91–94], though additional studies showed that MSCs have an inhibitory effect on tumor growth and metastasis [95, 96]. In addition to MSCs, MSC-EVs have a dual role in the progression of tumor cells which is related to the balance between inhibitory (e.g., miR-221, -23b, -1587) and promotional (e.g., miR-145, -124a, -16) bioactive molecules [97, 98]. MSCs derived from various sources such as adipose tissue, bone marrow, and dental pulp have the potency of bone formation in ectopic tissues [99, 100]. Therefore, ectopic differentiation of MSCs can be a severe barrier to MSC therapy in HSCT.

Another MSC-based therapy problem encountered is genetic instability. Chromosomal anomalies were reported at high frequency in long-term cultured mesenchymal stem cells [101]. In addition, it has been illustrated that malignant transformation can occur in MSCs [102]. Xiangrong Cui et al. reported IL22RA1/STAT3 signaling pathway plays a critical function in the malignant transformation of rat MSCs [103]. Malignant transformation of MSCs depends on MSC source (primary or tumoral), passage number, expansion protocol, and contamination of the cell culture media [104–106]. From a clinical point of view, regular genomic monitoring focusing on genomic stability is highly recommended before MSCs are used for clinical applications because the transplantation of elderly MSCs is less effective [102]. To date, no study has reported genetic instability and malignant transformation of MSC-EVs, which seem to be a good alternative for MSC-based therapy in regenerative medicine.

GVHD is one of the severe complications after allogenic-HSCT but seldom after transfusions or solid organ transplantations. GVHD affects 40–60% of all-HSCT recipients and accounts for 15% of deaths following HSCT [107]. MSCs amend GVHD because they have immunomodulatory properties via modulation of both innate and acquired immune pathways [108]. The excessive and prolonged suppression of the immune system by MSCs increases recipient susceptibility to opportunistic infections, but MSC-EVs with low immunosuppressive potency have no infection risks [3]. MSC-EV-derived miRNA-223, miRNA-564, and miRNA-451 regulate immune responses, hence having an inhibitory function in GVHD [64]. Ke-Liang et.al surveyed the effect of exosomes derived from a human bone marrow mesenchymal stem cell (hBMSC) on acute graft-versus-host disease (aGVHD) following allogeneic HSCT. They found that hBMSC-derived exosomes can reduce GVHD damages and increase the survival rate of aGVHD mice by altering the proportion of dendritic cells (DCs) and T cell subpopulation, as well as, preventing inflammatory responses in aGVHD mice [109]. Another study indicated that MSC-EVs derived from the human umbilical cord manage immune responses and prevent acute GVHD (aGVHD) via four mechanisms: (1) suppressing the proliferation of allo-responsive T cells, (2) altering the proportion of T cell subtypes, (3) inhibiting the release of several pro-inflammatory cytokines, e.g., IL-2, TNF-α and IFN-γ, and (4) inducing the release of anti-inflammatory cytokines, including IL-10 [110].

Thrombogenic risk of MSCs has also been demonstrated. MSCs exhibit tissue factor (TF) that in contact with blood initiates coagulation cascade after intravascular infusion of MSCs. In addition, TF has various functions in adhesion, migration, inflammation, and cell signaling, which are crucial for angiogenesis and tumor development [111]. Another study showed that TF and other proteins (e.g., coagulation factor V, prothrombin, myosin-9, histones, and CD9) in MSCs/MSC-EVs have pro coagulation activity [112] (see Table 1).

**Table 1:** Comparison between MSCs and MSC-EVs

Characteristic	MSCs	MSC-EVs	Notes	References
Determination of HSC fate	Yes	Similar to MSCs	Both MSCs and MSC-EVs have the proliferation and differentiation capacity of HSCs in vivo and in vitro*,* as well as inhibition of HSC apoptosis	[62, 73–75]
Malignant transformation	Yes/no	Not reported	Malignant transformation of MSCs depend on source, passage number, expansion protocol, medium conditions, etc	[91–96, 102]
Bone regeneration	Yes	Similar to MSCs	MSCs engaged in bone regeneration via differentiation to osteoblasts, MSC-EVs promote bone regeneration via microRNAs, especially *miR-335*	[3, 89–91]
Genetically instability	Possible	Not reported	Chromosomal anomalies in MSCs were seen at higher passages	[101]
Ectopic differentiation	Yes	Not reported	Bone formation in ectopic tissues after systemic infusion of MSCs were seen but not in MSC-EVs injection	[99, 100]
Opportunistic infections	High risk	Safe	MSCs are good vectors for microorganisms such as B19, CMV, HSV-1, and *Mycoplasma hyorhinis* but not reported for MSC-EVs	[85–87]
Immunosuppressive potency	Potent	Low potent	Increase recipient susceptibility to opportunistic infections	[3, 20, 21]
Risk of GVHD	Low Risk	Less than MSCs	Due to altering the proportion of immune cells, increasing the production of anti-inflammatory cytokines and decrease pro-inflammatory cytokine release	[64, 110]
Potential of tumor promoting effects	Dual role	Similar to MSCs	It depends on balance between inhibitory (e.g., *miR-221*, *-23b*, *-1587*) and promotional (e.g., *miR-145*, *-124a*, *-16*) bioactive molecules	[96–98]

MSCs: Mesenchymal stem cells; MSC-EVs: Mesenchymal stem cell-derived extracellular vesicles; HSC: Hematopoietic stem cell; miR: microRNA; GVHD: graft-versus-host disease; B19: *Parvovirus B19*; CMV*: Cytomegalovirus*; HSV-1: *Herpes Simplex*

Due to the ease of isolation and in vitro expansion of MSCs, proliferation and differentiation potency of HSCs, combined with their intriguing immunomodulatory properties and their impressive record of safety in a wide variety of clinical trials, it seems to be a good option for HSCT; hence, some disadvantages such as ectopic differentiation, malignant transformation, and opportunistic infections have limited the application of them. MSC-EVs simulate the effects of MSCs, but those are safe and do not have disadvantages of MSCs, hence making these derivatives a promising replacement for MSCs in HSCT.

## Conclusions and future perspectives

MSC-EVs affect the fate of the HSCs in the bone marrow microenvironment. MSC-EVs show similar effects to MSCs because they contain a variety of‏ growth factors, especially miRNAs. MSC-EVs have a crucial role in repairing cells as a new idea in cell-free therapy. These vesicles have the potential to alter the proliferation and differentiation of various cell types. It has also been proven that MSC-EVs play an important intercellular role in the proliferation and differentiation of HSCs. Hence, MSC-EVs can regulate the M/E ratio and suppress GVHD following HSCT. MSC-EVs have immunomodulatory properties that reduce the risk of GVHD. In addition, researchers demonstrated that MSC-EVs has no jeopardy of genetic instability, malignant transformation, and ectopic differentiation. Also, there is no evidence that MSC-EVs can transmit opportunistic infections. Therefore, MSC-EVs could be applied effectively as an adjuvant for HSCT instead of MSCs in the future. The possible advantage of EVs over MSCs and the possibility of EVs separation from different sources of MSCs in vitro get hopes up to establish an MSC-EV bank in the future. Hence, pretransplantation preconditioning regimens of MSC-EVs are essential and must be optimized for allogeneic HSCT. Further research should clarify the effect of preconditioned MSC-EVs on HSC biology in animal models of hematological diseases.

## Data Availability

Not applicable.

## Abbreviations

MSC: Mesenchymal stem cell
HSC: Hematopoietic stem cell
GVHD: Graft-versus-host disease
BM: Bone marrow
EVs: Extracellular vesicles
HSCT: Hematopoietic stem cell transplantation
BM-MSC: Bone marrow-derived mesenchymal stem cell
SDF1: Stromal cell-derived factor 1
CXCL12: C-X-C Motif Chemokine Ligand 12
miRNA: MicroRNA
M/E: Myeloid to erythroid precursors
IDO: Indoleamine 2,3-dioxygenase
HPCs: Hematopoietic progenitor cells
